# Lipid accumulation product and gallstone risk in US adults: A cross-sectional analysis of NHANES 2017–2020 data

**DOI:** 10.1371/journal.pone.0315235

**Published:** 2024-12-05

**Authors:** Xiaocheng Li, Jun Wang, Kai Leng

**Affiliations:** Department of Hepatopancreatobiliary Surgery, Third Affiliated Hospital of Zunyi Medical University (The First People’s Hospital of Zunyi), Zunyi, Guizhou, China; King’s College Hospital NHS Trust: King’s College Hospital NHS Foundation Trust, UNITED KINGDOM OF GREAT BRITAIN AND NORTHERN IRELAND

## Abstract

**Background:**

Abdominal obesity and insulin resistance are key risk factors for gallstones. The Lipid Accumulation Product (LAP), which combines waist circumference and triglyceride levels, may be a superior marker for visceral fat accumulation. However, its association with gallstone risk is unclear.

**Methods:**

Data from 3294 participants in the National Health and Nutrition Examination Survey (NHANES) 2017–2020 cycle were analyzed. Weighted logistic regression, subgroup analyses, and restricted cubic spline (RCS) analysis explored the relationship between LAP and gallstone risk. ROC analysis, along with Random Forest and CatBoost models, compared the predictive abilities of LAP with BMI.

**Results:**

Weighted logistic regression analysis showed that each unit increase in Ln-LAP was associated with a 52% higher risk of gallstones (OR: 1.52; 95% CI: 1.24–1.86; P = 0.009). Compared to the lowest tertile (T1), the second tertile (T2) had a higher risk of gallstones (OR: 1.76; 95% CI: 1.09–2.84; P = 0.082), and the third tertile (T3) had an even higher risk (OR: 2.27; 95% CI: 1.47–3.49; P = 0.021). RCS analysis showed a nonlinear positive relationship between Ln-LAP and gallstone risk (non-linear P < 0.001). Subgroup analyses indicated that Ln-LAP was significantly positively associated with the risk of gallstones in most subgroups, with no significant interactions observed among the subgroups. Weighted logistic regression analysis revealed a significant positive association between BMI ≥ 30 kg/m^2^ (obesity) and the risk of gallstones. ROC analysis indicated that Ln-BMI is a better predictor of gallstone risk than Ln-LAP. However, in Random Forest and CatBoost models, LAP exhibited predictive value similar to BMI for gallstone risk.

**Conclusion:**

While LAP is significantly and positively associated with an increased risk of gallstones, BMI generally appears to be a stronger predictor. However, LAP may still serve as a comparable marker under specific modeling conditions. Further research is needed to explore the relationship between LAP and gallstone risk.

## Introduction

Gallstones are a prevalent biliary disease affecting approximately 10–20% of the global population, leading to significant morbidity and healthcare costs [1–3]. The pathogenesis of gallstones is multifactorial, involving genetic predisposition, metabolic disturbances, dietary habits, and lifestyle factors [4]. Obesity and metabolic syndrome are well-established risk factors for gallstones. Traditionally, Body Mass Index (BMI) has been used to assess obesity and predict gallstone risk [5]. However, BMI’s limitations in differentiating fat distribution and metabolic health are well recognized.

The Lipid Accumulation Product (LAP), which integrates waist circumference (WC) and triglyceride (TG) levels, has emerged as a potentially superior marker for visceral fat accumulation and metabolic status [6]. Extensive research has demonstrated LAP’s high sensitivity and specificity in predicting metabolic syndrome and cardiovascular diseases [7–9]. Furthermore, some studies have found that LAP is more advantageous than traditional BMI in predicting type 2 diabetes [10, 11]. Additionally, a Japanese study highlighted LAP’s superiority over BMI in predicting metabolically associated fatty liver disease (MAFLD) [12]. Conversely, a Chinese study suggested that BMI might be a better predictor than LAP for MAFLD [13]. Despite the growing evidence supporting LAP’s effectiveness in various metabolic disorders, its role in evaluating gallstone risk remains minimally underexplored. Given the close association between metabolic disorders and gallstones, this gap in research underscores the need for further investigation into LAP’s potential utility in this area.

This study investigates the association between LAP and gallstone prevalence using National Health and Nutrition Examination Survey (NHANES) 2017–2020 data. Weighted logistic regression, subgroup analyses, and restricted cubic spline (RCS) analysis will explore this relationship. Additionally, ROC analysis, along with Random Forest and CatBoost models, will compare the predictive abilities of LAP and BMI for gallstone risk.

## Materials and methods

### Research design and participants

The National Center for Health Statistics (NCHS) oversees the NHANES, which collects representative data from both adults and children through health interviews, physical examinations, and laboratory tests. The program received ethical approval from the NCHS review board, and informed consent was obtained from all participants. For more information and data access, please visit the NHANES website (https://www.cdc.gov/nchs/nhanes/index.htm).

**Fig 1:**
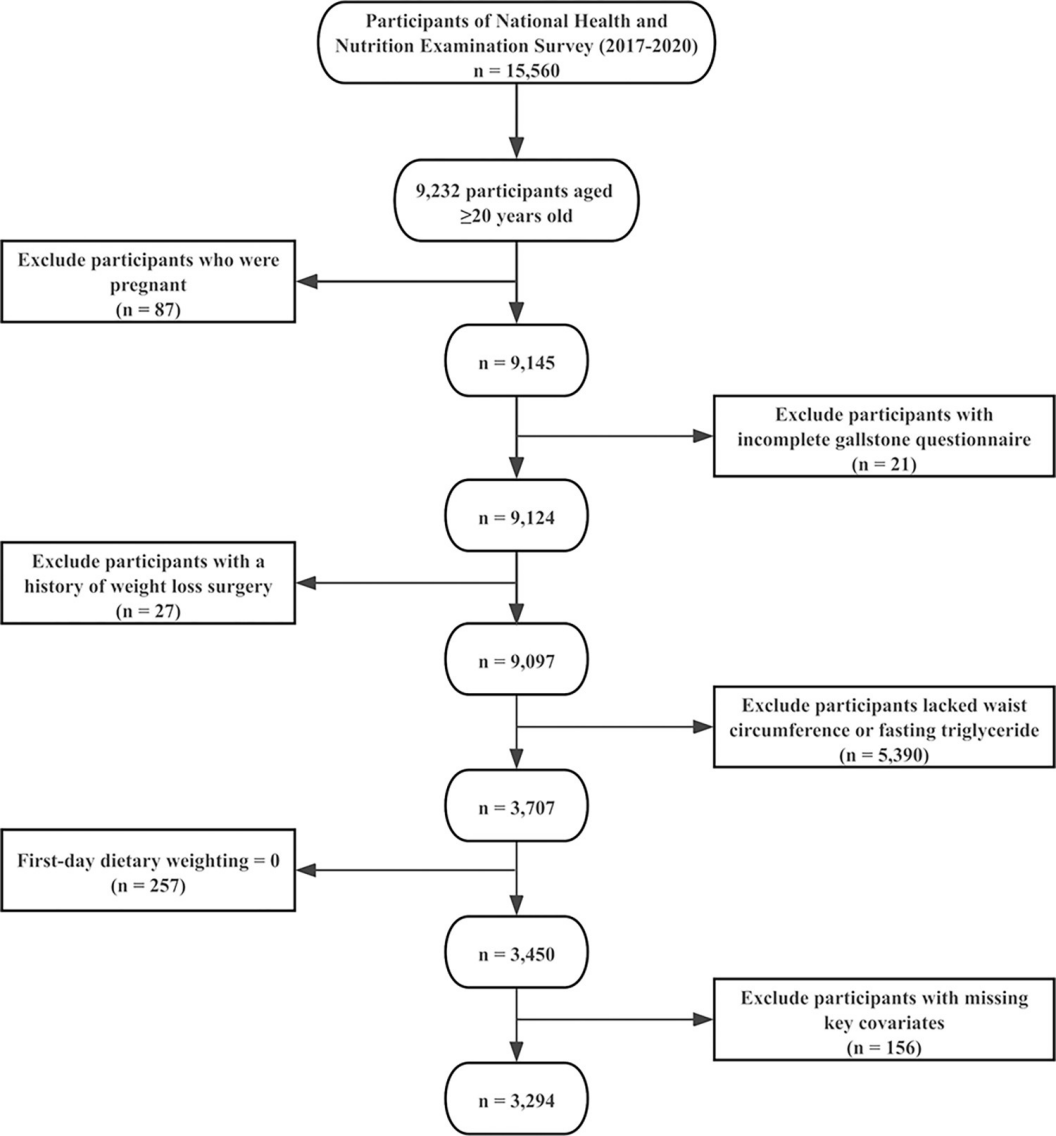
Participant selection flowchart. NHANES 2017–2020 initially enrolled 15,560 participants. After applying the inclusion and exclusion criteria, a total of 3294 participants were retained for the final analysis.

In the 2017–2020 cycle, participants were specifically asked to provide information on their history of gallstones. Initially, 15,560 participants were enrolled; however, 6,328 of them, under the age of 20, did not participate in the gallstone questionnaire. Additionally, 87 were pregnant, 21 refused to answer or were uncertain about their answers to questions regarding gallstones, 27 individuals had a history of weight loss surgery, 5390 lacked completed WC or TG levels, 257 had first-day dietary sampling weights of zero, and 156 had incomplete invariants. Therefore, these individuals were excluded from the study, resulting in a final sample size of 3294 participants. Fig 1 presents a flowchart of the selection process, and S1 Table in the S1 File details the missing data rates for key covariates.

### Definition of gallstone

To determine the presence of gallstones, participants were asked the question: "Has a doctor ever told you that you have gallstones?" Those who responded "Yes" were classified as having gallstones, while those who responded "No" were classified as not having gallstones.

### Calculation of LAP and BMI

In NHANES, anthropometric assessments and fasting blood tests were conducted by trained health technicians at the mobile examination center. Specifically, WC was measured with a tape measure at the upper edge of the iliac crest in centimeters (cm). The following formulas were used to calculate the LAP score: [WC (cm)– 65] × [TG (mmol/L)] for men and [WC (cm)– 58] × [TG (mmol/L)] for women.

BMI was calculated using weight and height measurements obtained by trained health technicians. Height was measured using a stadiometer to the nearest 0.1 cm, and weight was measured using a digital scale to the nearest 0.1 kg. BMI was defined as weight in kilograms divided by height in meters squared. In this study, participants were classified into the following BMI categories: normal weight (< 25.0 kg/m^2^), overweight (25.0–29.9 kg/m^2^), and obesity (≥ 30.0 kg/m^2^).

### Identification of covariates

The statistical model incorporated the following variables as covariates based on previous research [14, 15]: age (years), sex (male, female), race (Non-Hispanic White, Non-Hispanic Black, other), level of education (less than college, college or above), alcohol intake, leisure time physical activity, total energy intake, total cholesterol intake, total dietary fiber intake, total omega-3 fatty acid intake, total monounsaturated fatty acid intake, total vitamin C intake, total caffeine intake, Cholesterol-lowering medication (yes/no), Triglyceride-lowering medication (yes/no), and history of smoking, diabetes, and hypertension.

Alcohol intake was defined based on the questionnaire ALQ121 "Past 12 months how often drink alcoholic beverage" and questionnaire ALQ131 "During the past 12 months, on those days that you drank alcoholic beverages, on average, how many drinks did you have?" Participants who reported drinking 12 or more drinks per year were classified as alcohol drinkers.

Diabetes was defined as having an HbA1c ≥ 6.5%, a self-reported diagnosis of diabetes, a history of antidiabetic medication use, or fasting blood glucose ≥ 7 mmol/L. Blood pressure was measured using an oscillometric method. Hypertension was defined as a mean systolic blood pressure of ≥ 140 mmHg, a mean diastolic blood pressure of ≥ 90 mmHg, or a self-reported diagnosis of hypertension.

Smoking was defined according to the SMQ020 questionnaire as having smoked at least 100 cigarettes in their lifetime. Age was categorized into three groups: 20–39, 40–59, and ≥ 60 years. Leisure time physical activity was quantified using the formula: leisure time physical activity (minutes*MET) = (total vigorous recreational activity time × 8) + (total moderate recreational activity time × 4).

Cholesterol-lowering medication was defined as the use of statins (e.g., atorvastatin, simvastatin), ezetimibe, or colesevelam. Triglyceride-lowering medication was defined as the use of fibrates (e.g., fenofibrate), omega-3 fatty acids, or niacin.

Dietary intake data were collected from the first-day 24-hour dietary recall interviews conducted as part of the NHANES survey. Nutrient estimates were derived using the United States Department of Agriculture’s Food and Nutrient Database for Dietary Studies, which provides detailed nutritional information for the foods reported by participants.

### Statistical methods

Two-sided statistical testing was used, with statistical significance set at P < 0.05. All analyses were performed using R version 4.4.0. The NHANES official website recommends the use of appropriate sampling weights for statistical analysis and provides detailed guidance on weight analysis. This study used the first-day dietary weighting ’WTDRD1PP’ because the covariates involved data from the first day of dietary intake. The weights provided in the dataset were analyzed using the survey package in R.

Due to the non-normal distribution of LAP and BMI, transformations were applied: Ln-LAP was created using the log(LAP + 0.8532 + 1) transformation, and Ln-BMI was created using the log(BMI) transformation. The weighted tertile cutoff was used to explore the impact of Ln-LAP at different levels on the risk of gallstones. Continuous variables are presented as weighted survey means and standard deviations, whereas categorical variables are presented as counts and weighted survey percentages. Baseline data were analyzed using weighted t-tests or weighted chi-square tests, depending on the data type.

Following the STROBE guidelines [16], three multivariable regression models were constructed. In Model 1, no adjustments were made for covariates. Model 2 was adjusted for sex, age, race, and level of education. Model 3 was adjusted for sex, age, race, education, alcohol intake, hypertension, diabetes, smoking, cholesterol-lowering medication, triglyceride-lowering medication, leisure time physical activity, total energy intake, total cholesterol intake, total dietary fiber intake, total omega-3 fatty acid intake, total monounsaturated fatty acid intake, total vitamin C intake, total caffeine intake. To evaluate multicollinearity, this study used the Generalized Variance Inflation Factor and applied a degrees-of-freedom adjustment method. To reduce the impact of multicollinearity, total energy intake, total dietary fiber intake, and total monounsaturated fatty acid intake were standardized using Z-score normalization (mean = 0, standard deviation = 1) and then transformed into two principal components (PC1 and PC2) through principal component analysis (PCA).

Subgroup analyses for all variables were conducted using weighted multivariate regression analysis. Additionally, interaction terms were added to the models using the log-likelihood ratio test to examine heterogeneity in associations across subgroups. RCS were used to identify the dose-effect relationship between Ln-LAP and gallstone risk.

Weighted logistic regression combined with ROC curve analysis was employed to compare the predictive abilities of Ln-LAP and Ln-BMI for gallstone incidence. The DeLong test was used to determine whether the difference in the area under the curve (AUC) between the models was statistically significant.

To further validate the predictive effectiveness of Ln-LAP, the S2 File includes comparative analyses using Random Forest and CatBoost models which are widely used in medical research [17, 18].

Random Forest: Developed by Breiman [19], Random Forest is an ensemble learning method that builds multiple decision trees during training. For classification tasks, it outputs the majority class, while for regression tasks, it calculates the average prediction from all trees. This method is advantageous due to its ability to handle large datasets with high dimensionality, its robustness to overfitting through bootstrap aggregation (bagging), and its capacity to estimate variable importance.

CatBoost: Short for Categorical Boosting, CatBoost is a gradient boosting algorithm developed by Prokhorenkova et al. [20]. It is specifically optimized to handle categorical data without extensive preprocessing. CatBoost reduces prediction shift and provides high accuracy with less parameter tuning, making it particularly suitable for datasets with many categorical features.

Both models were implemented using their respective R packages, `randomForest`and `catboost`, with moderate parameter settings. Model performance was evaluated using ROC curve analysis, and predictive accuracy was assessed through the AUC metric.

### Ethical approval and consent to participate

The NHANES database was publicly accessible and all participants provided informed consent. Researchers can freely download and utilize relevant data for research and publication.

## Results

### Baseline characteristics

Table 1 summarizes the characteristics of the study population based on the tertiles of Ln-LAP, adjusted for survey weight. Females comprised 49.9% of the study group. Significant differences among the weighted Ln-LAP tertile groups were observed in terms of age, race, education level, history of alcohol intake, smoking history, Ln-BMI, history of diabetes, history of hypertension, leisure time physical activity, cholesterol-lowering medication and triglyceride-lowering medication (P < 0.05).

**Table 1:** Demographic and clinical characteristics by Ln-LAP tertiles from the National Health and Nutrition Examination Survey (NHANES) 2017–2020.

Characteristics	Overall	Tertiles of Ln-LAP			P-value
		T1	T2	T3	
	3294	1076	1146	1072	
**Sex**, n (%)					0.415
Male	1631 (50.1)	521 (47.1)	586 (51.8)	524 (51.3)	
Female	1663 (49.9)	555 (52.9)	560 (48.2)	548 (48.7)	
**Age**, n (%)					<0.001
20–39	1008 (36.5)	490 (53.8)	289 (29.3)	229 (26.5)	
40–59	1117 (34.4)	304 (25.8)	394 (35.2)	419 (42.1)	
≥60	1169 (29.1)	282 (20.3)	463 (35.6)	424 (31.4)	
**Race**, n (%)					<0.001
Non-Hispanic White	1183 (62.2)	347 (59.2)	383 (62.0)	453 (65.5)	
Non-Hispanic Black	827 (11.3)	341 (15.1)	309 (11.7)	177 (7.2)	
Other	1284 (26.5)	388 (25.8)	454 (26.3)	442 (27.3)	
**Education**, n (%)					0.009
Less than college	1386 (37.4)	404 (33.5)	480 (34.9)	502 (43.7)	
College or above	1908 (62.6)	672 (66.5)	666 (65.1)	570 (56.3)	
**Alcohol intake**, n (%)					0.048
No	1600 (41.1)	475 (38.9)	557 (39.1)	568 (45.2)	
Yes	1694 (58.9)	601 (61.1)	589 (60.9)	504 (54.8)	
**Smoking**, n (%)					0.025
No	1852 (56.0)	648 (60.5)	664 (57.6)	540 (49.7)	
Yes	1442 (44.0)	428 (39.5)	482 (42.4)	532 (50.3)	
**Diabetes**, n (%)					<0.001
No	2553 (84.3)	984 (94.9)	912 (87.6)	657 (70.5)	
Yes	741 (15.7)	92 (5.1)	234 (12.4)	415 (29.5)	
**Hypertension**, n (%)					<0.001
No	1803 (61.2)	769 (77.1)	594 (60.6)	440 (45.9)	
Yes	1491 (38.8)	307 (22.9)	552 (39.4)	632 (54.1)	
**Cholesterol-lowering medication**, n (%)					<0.001
No	2535 (79.0)	925 (87.2)	882 (80.1)	728 (69.7)	
Yes	759 (21.0)	151 (12.8)	264 (19.9)	344 (30.3)	
**Triglyceride-lowering medication**, n (%)					0.018
No	3248 (98.3)	1066 (98.8)	1136 (99.2)	1046 (96.7)	
Yes	46 (1.7)	10 (1.2)	10 (0.8)	26 (3.3)	
**Ln-BMI**	3.36 (0.23)	3.18 (0.16)	3.37 (0.16)	3.53 (0.20)	<0.001
**Leisure time physical activity**, mean (SD), minutes*MET	968.11 (1624.75)	1295.51 (1948.35)	1000.52 (1625.74)	607.96 (1116.13)	<0.001
**Total energy intake**, mean (SD), kcal	2148.21 (928.31)	2116.74 (933.63)	2122.35 (886.74)	2205.58 (961.23)	0.102
**Total dietary fiber intake**, mean (SD), gm	16.72 (10.57)	17.52 (11.54)	16.57 (10.49)	16.06 (9.53)	0.148
**Total cholesterol intake**, mean (SD), mg	310.56 (242.63)	306.44 (250.58)	311.14 (236.94)	314.09 (240.31)	0.893
**Total omega-3 fatty acid intake**, mean (SD), gm	0.11 (0.34)	0.12 (0.37)	0.10 (0.30)	0.12 (0.35)	0.444
**Total monounsaturated fatty acids intake**, mean (SD), gm	29.65 (16.54)	29.41 (17.47)	29.48 (15.25)	30.07 (16.82)	0.806
**Total vitamin C intake**, mean (SD), mg	75.66 (86.09)	80.31 (81.33)	75.14 (91.04)	71.53 (85.46)	0.219
**Total caffeine intake**, mean (SD), mg	172.91 (224.33)	163.54 (260.20)	170.12 (199.70)	185.08 (207.89)	0.272

**Abbreviations:** BMI, body mass index; SD, standard deviation; LAP, lipid accumulation product; MET, metabolic equivalent of task.

Means, percentages, and Ln-LAP tertiles were adjusted for the survey weights of the National Health and Nutrition Examination Survey (NHANES).

### Association between Ln-LAP and gallstone risk

The association between Ln-LAP and the risk of gallstones was examined using three different models, adjusting for various confounders. The results were presented in Table 2.

**Table 2:** Association between Ln-LAP and gallstone risk.

Exposure	Model 1		Model 2		Model 3	
	OR (95% CI)	P-value	OR (95% CI)	P-value	OR (95% CI)	P-value
**Ln-LAP (continuous)**	1.77 (1.51, 2.06)	<0.001	1.69 (1.40, 2.03)	<0.001	1.52 (1.24, 1.86)	0.009
**Tertile of Ln-LAP**						
T1, [0–3.33]	Ref.		Ref.		Ref.	
T2, [3.33–4.08]	2.14 (1.31, 3.50)	0.006	1.84 (1.16, 2.92)	0.019	1.76 (1.09, 2.84)	0.082
T3, [4.08–7.21]	3.23 (2.13, 4.92)	<0.001	2.76 (1.78, 4.26)	<0.001	2.27 (1.47, 3.49)	0.021
P for trend		<0.001		<0.001		<0.001

Model 1: not adjusted.

Model 2: adjusted for sex, age, race, education.

Model 3: adjusted for sex, age, race, education, alcohol intake, hypertension, diabetes, smoking, leisure time physical activity, cholesterol-lowering medication, triglyceride-lowering medication, PC1, PC2, total cholesterol intake, total omega-3 fatty acid intake, total vitamin C intake and total caffeine intake. PC1 and PC2 were two principal components derived through principal component analysis from total energy intake, total dietary fiber intake, and total monounsaturated fatty acids intake.

Results were adjusted for the survey weights of the National Health and Nutrition Examination Survey (NHANES).

**Abbreviations:** LAP, lipid accumulation product; OR, odds ratio; CI, confidence interval.

In Model 3, after adjusting all covariables, each unit increase in Ln-LAP was associated with a 52% higher risk of gallstones (OR: 1.52; 95% CI: 1.24, 1.86; P < 0.001).

#### Ln-LAP tertiles

Participants in the second tertile (T2) showed a trend towards a higher risk of gallstones compared with the reference group (T1). The OR for T2 was 1.76 (95% CI: 1.09, 2.84; P = 0.082) in Model 3.

The third tertile (T3) exhibited an even higher risk, with OR of 2.27 (95% CI: 1.47, 3.49; P = 0.021) in Model 3.

The GVIFs from the weighted logistic regression between Ln-LAP tertiles and gallstone risk, adjusted for all covariates in Model 3, were presented in S2 Table of S1 File. All GVIFs were less than 10, and most are below 5, indicating that multicollinearity was not a significant concern.

The risk trend was statistically significant across all models (P for trend < 0.001).

**Fig 2:**
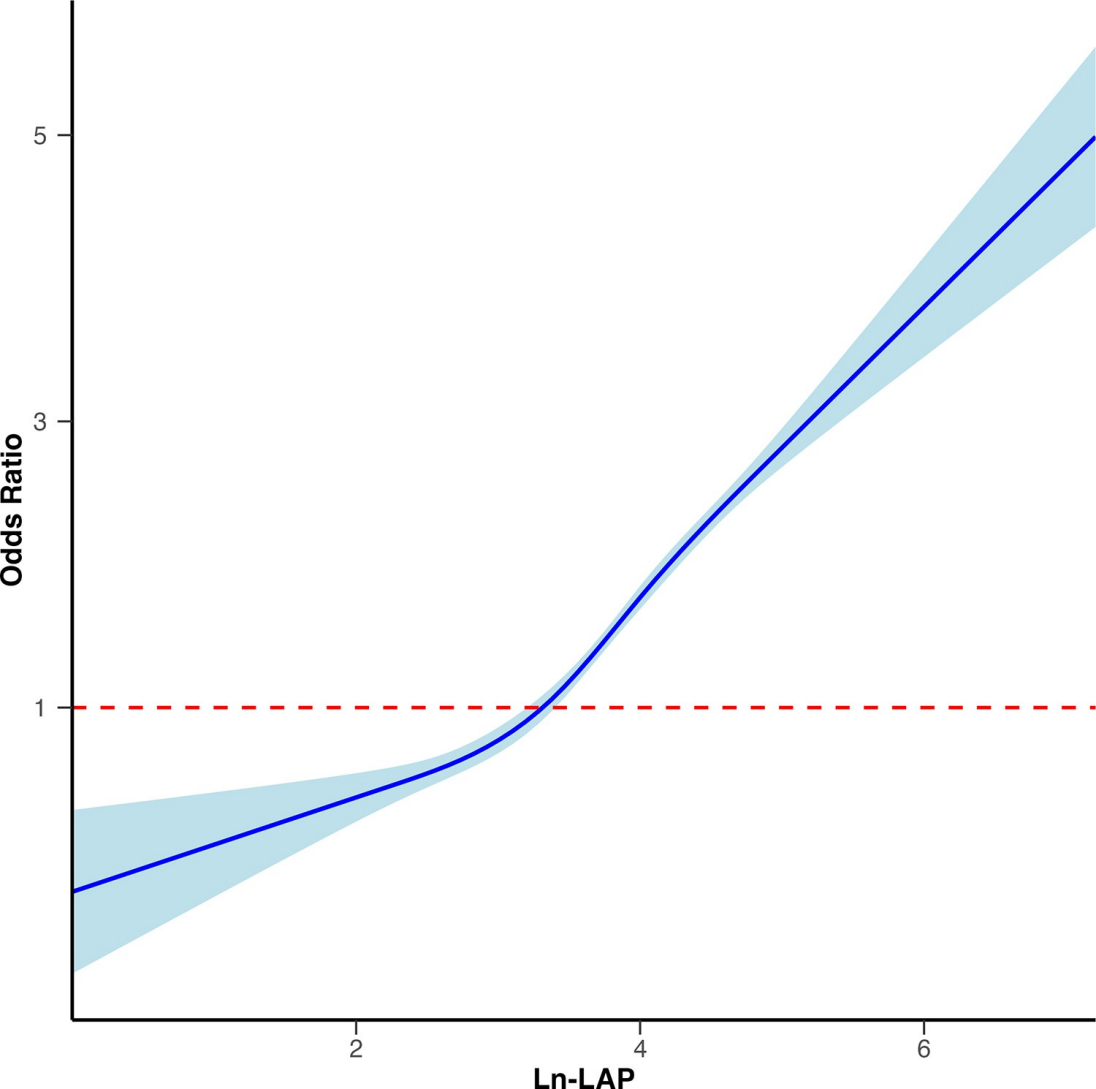
Nonlinear positive relationship between Ln-LAP and gallstone risk, as shown by RCS analysis.

Smoothed curve fitting by the RCS further displayed a nonlinear positive relationship between Ln-LAP and gallstone risk (non-linear P < 0.001; Fig 2).

### Subgroup analyses

Subgroup analysis revealed a positive association between higher levels of Ln-LAP and the risk of gallstones in several clinical categories (Table 3). Significant associations were observed in younger adults aged 20–39 years, females, non-Hispanic Whites, and the ’Other’ racial category. Additionally, those with less than a college education, individuals with hypertension, non-diabetics, alcohol consumers, and participants not taking cholesterol-lowering medication showed a significant positive correlation between higher Ln-LAP and gallstone risk (P < 0.05). However, no significant interactions were observed between subgroups. These findings suggest that increased Ln-LAP levels are consistently associated with a higher risk of gallstones across various subgroups.

**Table 3:** Subgroup analyses for the association between Ln-LAP and gallstone risk.

Subgroups	Tertiles of Ln-LAP			P for interaction
	T1	T2	T3	
	Ref	OR (95%CI) P-value	OR (95%CI) P-value	
**Age (years)**				0.064
20–39	1.00	1.35 (0.39, 4.67) 0.654	**4.84 (2.16, 10.86) 0.009**	
40–59	1.00	1.84 (0.76, 4.43) 0.224	1.49 (0.66, 3.35) 0.374	
≥60	1.00	1.71 (0.72, 4.07) 0.272	2.28 (1.04, 5.00) 0.087	
**Sex**				0.507
Male	1.00	1.46 (0.60, 3.56) 0.441	1.64 (0.51, 5.24) 0.442	
Female	1.00	1.84 (1.07, 3.16) 0.078	**2.44 (1.33, 4.50) 0.035**	
**Race**				0.628
Non-Hispanic White	1.00	1.89 (0.96, 3.70) 0.114	**2.22 (1.37, 3.58) 0.017**	
Non-Hispanic Black	1.00	1.30 (0.45, 3.74) 0.673	2.93 (0.75, 11.51) 0.263	
Other	1.00	1.59 (1.05, 2.41) 0.073	**2.34 (1.23, 4.47) 0.041**	
**Education**				0.237
Less than college	1.00	2.93 (1.21, 7.08) 0.063	**3.29 (1.48, 7.29) 0.033**	
College or above	1.00	1.42 (0.80, 2.49) 0.282	2.13 (1.18, 3.83) 0.053	
**Hypertension**				0.333
Yes	1.00	**2.50 (1.35, 4.65) 0.034**	**3.12 (1.71, 5.68) 0.014**	
No	1.00	1.42 (0.87, 2.32) 0.224	1.98 (1.07, 3.65) 0.081	
**Diabetes**				0.499
Yes	1.00	3.39 (1.06, 10.87) 0.095	3.50 (1.19, 10.35) 0.073	
No	1.00	1.57 (0.98, 2.51) 0.122	**2.12 (1.37, 3.27) 0.020**	
**Alcohol intake**				0.190
Yes	1.00	1.28 (0.55, 2.97) 0.592	**2.59 (1.31, 5.15) 0.042**	
No	1.00	2.35 (1.20, 4.60) 0.056	2.09 (1.15, 3.78) 0.059	
**Smoking**				0.702
Yes	1.00	2.29 (0.84, 6.24) 0.166	2.81 (1.08, 7.34) 0.088	
No	1.00	1.45 (0.83, 2.52) 0.244	2.01 (1.13, 3.57) 0.064	
**Cholesterol-lowering medication**				0.262
Yes	1.00	0.84 (0.26, 2.72) 0.783	2.05 (0.81, 5.17) 0.191	
No	1.00	**2.27 (1.29, 4.02) 0.037**	**2.33 (1.36, 4.01) 0.028**	

Results were adjusted for sex, age, race, education, alcohol intake, hypertension, diabetes, smoking, leisure time physical activity, cholesterol-lowering medication, triglyceride-lowering medication, PC1, PC2, total cholesterol intake, total omega-3 fatty acid intake, total vitamin C intake and total caffeine intake, except the variable itself. PC1 and PC2 were two principal components derived through principal component analysis from total energy intake, total dietary fiber intake, and total monounsaturated fatty acids intake. All analyses were adjusted for the survey weights of the National Health and Nutrition Examination Survey (NHANES).

**Abbreviations:** LAP, lipid accumulation product; OR, odds ratio; CI, confidence interval.

### Comparison of LAP and BMI in predicting gallstone risk

The results of the weighted multivariable logistic regression indicated a positive association between obesity (BMI ≥ 30 kg/m^2^) and the risk of gallstones after adjusting for all covariates (S3 Table in S1 File).

**Fig 3:**
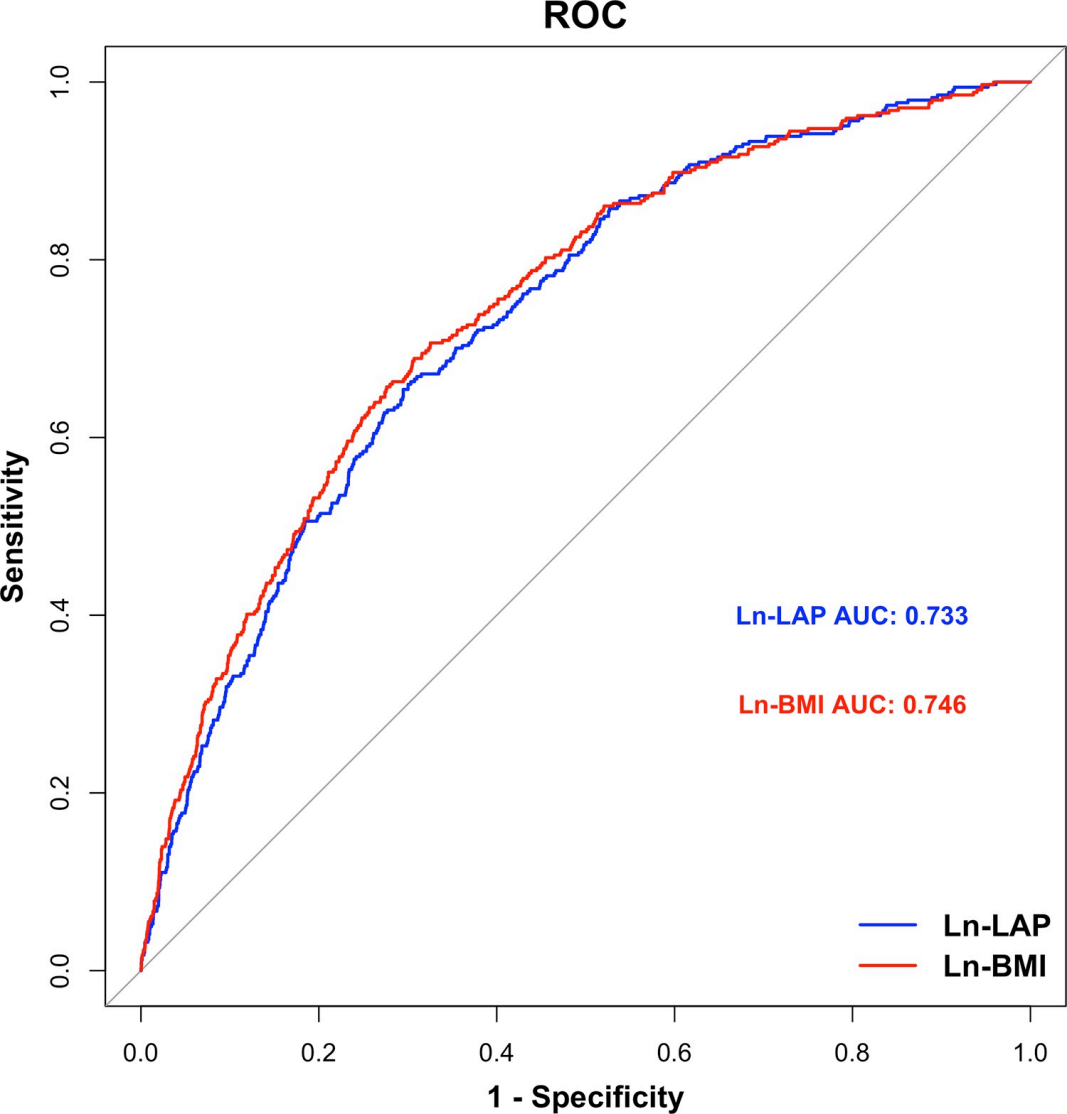
ROC curves for Ln-LAP and Ln-BMI prediction of gallstone risk.

The results of the ROC curves were shown in Fig 3, and a detailed comparison between Ln-LAP and Ln-BMI in predicting gallstone risk using weighted logistic regression was available in S4 Table in S1 File. ROC analysis demonstrated that Ln-BMI (AUC = 0.746) had a better predictive value for gallstones compared to Ln-LAP (AUC = 0.733). Delong’s test confirmed that the difference between the AUC values of Ln-LAP and Ln-BMI was statistically significant (P  =  0.018), indicating that Ln-BMI is superior to Ln-LAP in predicting gallstones.

The Random Forest and CatBoost models demonstrated that LAP had a predictive ability comparable to BMI in predicting the risk of gallstones. DeLong’s test indicated no significant difference in AUC values between Ln-BMI and Ln-LAP. Detailed performance metrics of the models were available in S2 File, with results presented in S1 Table and S1 Fig within the same file.

## Discussion

This study utilized NHANES 2017–2020 data, analyzing a total of 3294 participants, and found a significant positive association between Ln-LAP and gallstone risk. Weighted logistic regression revealed that, after adjusting for all covariates, each unit increase in Ln-LAP was associated with a 52% higher risk of gallstones (P < 0.05). Compared to the first tertile (T1), participants in the third tertile (T3) exhibited a significantly higher gallstone risk (P < 0.05). The smoothed curve fitting showed a nonlinear positive relationship between Ln-LAP and gallstone risk (non-linear P < 0.001). Subgroup analysis indicated a positive association between higher levels of Ln-LAP and gallstone risk across various demographic and clinical categories. These findings underscore the potential role of LAP as a valuable marker for assessing gallstone risk. Additionally, this study also found that BMI was also positively correlated with gallstone risk, particularly when considering obesity (BMI ≥ 30kg/m^2^). ROC analysis demonstrated that Ln-BMI outperformed in predicting gallstone risk. However, the Random Forest and CatBoost models demonstrated similar overall predictive performance for both markers, consistent with some of the findings in logistic regression analysis. This consistency suggests robustness in the conclusion that BMI is an important predictor of gallstone risk, while also indicating that LAP remains a viable alternative under certain modeling approaches.

The potential mechanisms underlying the positive association between LAP and gallstone risk could be linked to insulin resistance, which is commonly observed in individuals with higher visceral fat accumulation [21–23]. LAP, as a composite index combining WC and TG levels, serves as a marker for abdominal obesity and lipid accumulation, both of which are linked to insulin resistance [24]. Hyperinsulinemia, a consequence of insulin resistance, can lead to increased hepatic cholesterol uptake and heightened cholesterol saturation in bile, thereby promoting gallstone formation. This mechanism aligns with previous studies suggesting that LAP is closely associated with various metabolic disorders, including non-alcoholic fatty liver disease and type 2 diabetes, which are also known risk factors for gallstones [25, 26]. Thus, despite the limited availability of prior studies directly investigating the association between LAP and gallstone risk, LAP has been previously associated with other metabolic conditions, which could provide indirect support for its relevance to gallstone formation.

Additionally, this study found that a BMI ≥ 30 kg/m^2^ was positively associated with an increased risk of gallstones, which is consistent with previous findings [27]. However, earlier studies have suggested that measures of abdominal obesity, such as WC and waist-to-hip ratio, may serve as better predictors of gallstone risk compared to BMI alone [24]. This discrepancy might be attributed to differences in the population, study design or models used for analysis. Moreover, the divergent predictive outcomes between traditional weighted logistic regression and the two machine learning models in this study could be influenced by the inherent distinctions in how these methods manage complex interactions and non-linear relationships within the data. Machine learning models, such as Random Forest and CatBoost, are capable of capturing subtle nuances and intricate interactions between variables that traditional logistic regression may not adequately address [28].

It is also important to consider that this study employed a cross-sectional design, which limits our ability to infer causation between LAP and gallstone risk. Although a robust association was observed, it cannot be concluded that elevated LAP directly causes gallstones. The cross-sectional nature of the data prevents establishing a temporal sequence that is necessary to definitively determine causality. Future longitudinal studies are needed to better understand the causal pathways among visceral fat, lipid accumulation, and gallstone formation. Moreover, intervention studies that focus on modifying LAP components, such as reducing WC or TG levels, could provide further insights into whether changes in LAP can directly influence gallstone risk.

This study had several strengths, including the use of a nationally representative dataset from NHANES, which enhances the generalizability of the findings to the broader U.S. population. Moreover, both traditional statistical methods and machine learning approaches, such as Random Forest and CatBoost models, were employed to validate the results, thereby improving their robustness. Careful adjustment for potential confounders further strengthened the credibility of the findings. However, certain limitations must be acknowledged. First, the reliance on self-reported data to assess gallstone status introduces potential recall bias, which may affect the accuracy of the results. Second, while numerous known confounders were adjusted for, the potential influence of unmeasured or unknown confounders cannot be entirely excluded. Lastly, given that NHANES data are representative of the U.S. population, the findings may not be directly applicable to other populations with different genetic, environmental, or dietary backgrounds.

## Conclusion

LAP is significantly and positively associated with gallstone risk, while BMI shows a better predictive ability of gallstone risk overall. Both indices can be useful depending on the method applied. Further research is necessary to explore these associations in more detail.

## Supporting information

S1 File Supplementary tables for covariates and gallstone risk analysis. (DOCX)

S2 File Machine learning comparison of LAP and BMI in predicting gallstone risk based on NHANES 2017–2020 data. (DOCX)
