# Supplementary material for: Lipid accumulation product and gallstone risk in US adults: A cross-sectional analysis of NHANES 2017–2020 data
Source: PLoS One. 2024 Dec 5;19(12):e0315235. doi: 10.1371/journal.pone.0315235 (PMC11620675; doi:10.1371/journal.pone.0315235)
Supplement: S1 File — (DOCX) [file pone.0315235.s001.docx]

**Supplementary tables for covariates and gallstone risk analysis**

**S1 Table.** Covariates missingness rates.

| **Covariates** | **Missing rate** |
| --- | --- |
| Sex | 0 |
| Race | 0 |
| Education | 0 |
| Age | 0 |
| Alcohol intake | 2.38% |
| Diabetes | 1.77% |
| Hypertension | 0 |
| Smoking | 0.06% |
| Leisure time physical activity | 0.09% |
| Cholesterol-lowering medication | 0 |
| Triglyceride-lowering medication | 0 |
| Total energy intake | 0 |
| Total dietary fiber intake | 0 |
| Total cholesterol intake | 0 |
| Total omega-3 fatty acid intake | 0 |
| Total monounsaturated fatty acids intake | 0 |
| Total vitamin C intake | 0 |
| Total caffeine intake | 0 |
| Ln-BMI | 0.29% |

**Abbreviations:** BMI, body mass index.

**S2 Table. GVIFs for covariates in weighted logistic regression model 3 assessing the association between Ln-LAP tertiles and gallstone risk.**

| **Covariates** | **GVIF** |
| --- | --- |
| Sex | 3.76 |
| Race | 1.99 |
| Education | 5.74 |
| Age | 2.6 |
| Alcohol intake | 5.62 |
| Diabetes | 3.33 |
| Hypertension | 2.23 |
| Smoking | 3.18 |
| Leisure time physical activity | 2.61 |
| Cholesterol-lowering medication | 2.89 |
| Triglyceride-lowering medication | 1.89 |
| PC1 | 5.52 |
| PC2 | 4.52 |
| Total cholesterol intake | 3.85 |
| Total omega-3 fatty acid intake | 4.12 |
| Total vitamin C intake | 4.27 |
| Total caffeine intake | 4.23 |
| Tertiles of Ln-LAP | 3.74 |

**Note:** PC1 and PC2 were two principal components derived through principal component analysis from total energy intake, total dietary fiber intake, and total monounsaturated fatty acids intake.

**Abbreviations:** GVIF, Generalized Variance Inflation Factor; LAP, lipid accumulation product.

**S3 Table. Association between BMI and gallstone risk after adjusting all covariates.**

| **Covariates** | **OR** | **95%CI** | **P** |
| --- | --- | --- | --- |
| Sex |  |  |  |
| Male | Ref. | Ref. | Ref. |
| Female | 2.97 | (1.95, 4.54) | 0.006 |
| Age |  |  |  |
| 20-39 | Ref. | Ref. | Ref. |
| 40-59 | 2.37 | (0.98, 5.75) | 0.057 |
| ≥60 | 2.83 | (1.34, 5.96) | 0.021 |
| Race |  |  |  |
| Non-Hispanic White | Ref. | Ref. | Ref. |
| Non-Hispanic Black | 0.33 | (0.22, 0.51) | 0.006 |
| Other | 1.17 | (0.79, 1.74) | 0.474 |
| Education |  |  |  |
| Less than college | Ref. | Ref. | Ref. |
| College or above | 1.11 | (0.81, 1.52) | 0.538 |
| Alcohol intake |  |  |  |
| No | Ref. | Ref. | Ref. |
| Yes | 0.74 | (0.51, 1.07) | 0.103 |
| Smoking |  |  |  |
| No | Ref. | Ref. | Ref. |
| Yes | 1.26 | (0.81, 1.96) | 0.306 |
| Diabetes |  |  |  |
| No | Ref. | Ref. | Ref. |
| Yes | 1.37 | (0.90, 2.09) | 0.141 |
| Hypertension |  |  |  |
| No | Ref. | Ref. | Ref. |
| Yes | 1.47 | (0.89, 2.43) | 0.137 |
| Cholesterol-lowering medication |  |  |  |
| No | Ref. | Ref. | Ref. |
| Yes | 0.81 | (0.53, 1.26) | 0.398 |
| Triglyceride-lowering medication |  |  |  |
| No | Ref. | Ref. | Ref. |
| Yes | 0.69 | (0.17, 2.87) | 0.632 |
| Leisure time physical activity | 1.00 | (1.00, 1.00) | 0.959 |
| PC1 | 1.11 | (0.98, 1.26) | 0.200 |
| PC2 | 1.06 | (0.80, 1.40) | 0.689 |
| Total cholesterol intake | 1.00 | (1.00, 1.00) | 0.336 |
| Total omega-3 fatty acid intake | 0.97 | (0.55, 1.73) | 0.935 |
| Total vitamin C intake | 1.00 | (1.00, 1.00) | 0.523 |
| Total caffeine intake | 1.00 | (1.00, 1.00) | 0.890 |
| BMI |  |  |  |
| Normal weight | Ref. | Ref. | Ref. |
| Overweight | 1.55 | (0.83, 2.91) | 0.225 |
| **Obesity** | **3.00** | **(1.35, 6.64)** | **0.008** |

**Note:** PC1 and PC2 were two principal components derived through principal component analysis from total energy intake, total dietary fiber intake, and total monounsaturated fatty acids intake.

**Abbreviations:** BMI, body mass index; OR, odds ratio; CI, confidence interval.

**S4 Table.** Comparison of ROC curves for Ln-LAP and Ln-BMI in predicting gallstone risk using weighted logistic regression.

|  | **AUC (95%CI)** | **Cutoff** | **Sensitivity** | **Specificity** |
| --- | --- | --- | --- | --- |
| Ln-LAP | 0.733 (0.707, 0.760) | 0.116 | 0.660 | 0.700 |
| Ln-BMI | 0.746 (0.719, 0.773) | 0.110 | 0.689 | 0.693 |

**Abbreviations:** LAP, lipid accumulation product; BMI, body mass index; AUC, area under the curve; CI, confidence interval.
