# Supplementary material for: Lipid accumulation product and gallstone risk in US adults: A cross-sectional analysis of NHANES 2017–2020 data
Source: PLoS One. 2024 Dec 5;19(12):e0315235. doi: 10.1371/journal.pone.0315235 (PMC11620675; doi:10.1371/journal.pone.0315235)
Supplement: S2 File — (DOCX) [file pone.0315235.s002.docx]

**Machine learning comparison of LAP and BMI in predicting gallstone risk based on NHANES 2017-2020 data**

**Introduction**

To further compare the predictive effectiveness of the lipid accumulation product (LAP) and body mass index (BMI) for gallstone incidence, Random Forest and CatBoost models were employed. This section provides a detailed description of the machine learning models, data processing methods, and the results obtained from these analyses. Due to the primary focus on comparing the predictive abilities of LAP and BMI for gallstone risk, extensive model tuning was not performed.

**Methods**

**Data Preprocessing**

The National Health and Nutrition Examination Survey (NHANES) 2017-2020 dataset, following the selection process outlined in Fig 1 of the main text, was used for this analysis. The data were preprocessed by converting categorical variables into numerical format and normalizing continuous variables. Covariates included sex, age, race, education, alcohol intake, hypertension, diabetes, smoking, cholesterol-lowering medication, triglyceride-lowering medication, leisure time physical activity, total energy intake, total cholesterol intake, total dietary fiber intake, total omega-3 fatty acid intake, total monounsaturated fatty acid intake, total vitamin C intake and total caffeine intake. Due to the non-normal distribution of LAP and BMI, logarithmic transformations were applied: Ln-LAP was created using the log(LAP + 0.8532 + 1) transformation, and Ln-BMI was created using the log(BMI) transformation.

**Model Training and Evaluation**

**Random Forest**

Random Forest models were trained using balanced datasets created by the Random Over-Sampling Examples (ROSE) method. Hyperparameter tuning was conducted using cross-validation. Key parameters included:

Number of trees (ntree): 500

Number of randomly selected features (mtry): integers between 2 and 10

Class weights: 7 for the positive class (gallstones), 1 for the negative class

Cross-validation control defined using the `trainControl` function, set to 5-fold cross-validation and utilizing the `twoClassSummary` function for evaluation metrics.

During the final model training, variable importance and the Out-of-Bag (OOB) error as a function of the number of trees were also evaluated.

**CatBoost**

CatBoost models were also trained using balanced datasets created by the ROSE method. Hyperparameter tuning was conducted using cross-validation. Key parameters included:

Number of iterations: 3000

Tree depth: 10

Learning rate: 0.01

L2 leaf regularization: 3

Custom and evaluation metrics: AUC

Class weights: 7 for the positive class (gallstones), 1 for the negative class

Cross-validation using `catboost.cv` with 5-fold validation to determine the optimal number of iterations.

The optimal number of iterations for both the Ln-LAP and Ln-BMI datasets were identified via cross-validation, with 855 iterations for Ln-LAP and 784 iterations for Ln-BMI. The final models were then retrained using these optimal iteration settings.

**Performance Evaluation**

Models were evaluated on a test set using predictions from the trained models. The steps included:

Calculating prediction probabilities and class predictions for both Random Forest and CatBoost models on the test set.

Utilizing the `roc` function to compute the receiver operating characteristic (ROC) curves and area under the curve (AUC) values.

Generating confusion matrices using the `confusionMatrix` function and extracting evaluation metrics such as accuracy, sensitivity, specificity, Kappa coefficient, and balanced accuracy.

**Performance Comparison**

The DeLong test was used to determine whether the difference in AUC between the models was statistically significant. A performance table was created to summarize key performance metrics for each model and was saved as an Excel file. The steps included:

Creating a summary table containing model names, AUC values, accuracy, sensitivity, specificity, Kappa coefficient, and balanced accuracy.

Using the `openxlsx` package to create an Excel workbook and save the performance comparison results.

Plotting and saving ROC curves for all models to visually compare their classification performance.

**Results**

The performance metrics, including AUC, accuracy, sensitivity, specificity, Kappa, and balanced accuracy, are summarized in S1 Table. The ROC curves are shown in S1 Fig.

In both the Random Forest and CatBoost models, Ln-LAP and Ln-BMI demonstrated very similar predictive capabilities. The AUC values for both features were nearly identical in each model, and the DeLong Test indicated that the differences were not statistically significant (P > 0.05). Sensitivity for Ln-BMI was slightly higher compared to Ln-LAP in both models, but this was accompanied by a minor decrease in specificity. Overall, the results suggest no significant difference between Ln-LAP and Ln-BMI in terms of predicting gallstone risk, indicating that both features are similarly effective.

These findings partially align with the main analysis results from the logistic regression model, which indicated that Ln-BMI had a higher predictive value compared to Ln-LAP. Therefore, while the machine learning models suggest that both features are comparable, the logistic regression model highlights that Ln-BMI may have a higher predictive value, particularly in identifying positive cases.

**S1 Table.** **Results of Random forest and CatBoost models comparing Ln-LAP with Ln-BMI in predicting gallstone risk.**

| **Model** | **AUC** | **Accuracy** | **Sensitivity** | **Specificity** | **Kappa** | **Balanced Accuracy** |
| --- | --- | --- | --- | --- | --- | --- |
| **Random Forest** |  |  |  |  |  |  |
| Ln-LAP | 0.683 | 0.669 | 0.676 | 0.603 | 0.135 | 0.640 |
| Ln-BMI | 0.682 | 0.688 | 0.705 | 0.544 | 0.129 | 0.625 |
| **CatBoost** |  |  |  |  |  |  |
| Ln-LAP | 0.664 | 0.661 | 0.675 | 0.544 | 0.107 | 0.609 |
| Ln-BMI | 0.670 | 0.672 | 0.685 | 0.559 | 0.121 | 0.622 |

**Abbreviations:** LAP, lipid accumulation product; BMI, body mass index; AUC, area under curve.

**
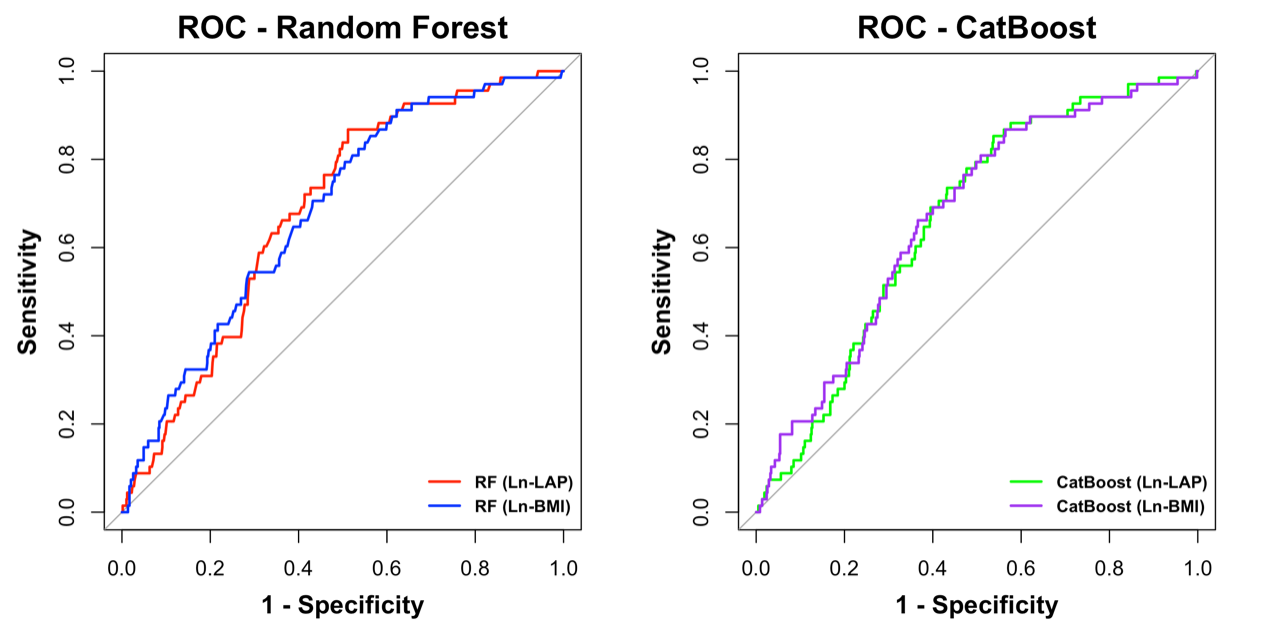
**

**S1 Fig.** **ROC curves for random forest and CatBoost model.**
